# Bone in osteogenesis imperfecta: features and therapy

**DOI:** 10.3389/fcell.2026.1950969

**Published:** 2026-10-29

**Authors:** Giacomina Brunetti

**Affiliations:** Department of Biosciences, Biotechnologies and Environment, University of Bari Aldo Moro, Bari, Italy

**Keywords:** bisphosphonates, cell therapy, collagen type I, gene therapy, osteogenesis imperfecta

## Abstract

Osteogenesis imperfecta (OI) is a heterogeneous group of inherited bone dysplasia, characterized by low bone mass and bone fragility with possible development of fractures. OI has been known as a predominantly inherited disease caused by mutations in either of the genes encoding type I collagen (*COL1A1* and *COL1A2*). Further studies in recent years have identified new mutations in collagen-related genes, leading to the identification and classification of different types of OI. Therapeutic approaches have concentrated on the use of bisphosphonates and/or monoclonal antibodies as elective therapy, but different trials are ongoing using cell and gene therapies, and these offer new options for OI patients.

## Introduction

A heterogeneous group of inherited bone dysplasia is represented by osteogenesis imperfecta (OI), also known as “brittle bone disease” (Marini et al., 2017)—a rare disease with an incidence of 1 in 15–20,000 births (Forlino and Marini, 2016). It is a genetically inherited disease characterized by low bone mass and bone fragility (Rauch and Glorieux, 2004), with consequent skeleton deformity and possible fractures due to generalized connective tissue disorganization (Blandin et al., 2025). Fracture incidence in OI is at a maximum during childhood and then declines in midlife, although this remains higher than in the general population (Liu et al., 2023). Fracture risk increases again in the elderly (Folkestad et al., 2017). In postmenopausal women and men over 55 years old with OI, fracture rates are 4.9- to 8.0-fold higher than those in non-OI individuals (Folkestad et al., 2017). Additional features include blue sclera, hearing loss, malocclusion, dentinogenesis imperfecta, cardiac valve abnormalities, pulmonary function alteration, ligament laxity, and muscle weakness (Mordenti et al., 2023).

Since the early 1980s, OI has been known as a predominantly inherited disease caused by mutations in either of the genes that encode type I collagen (*COL1A1* and *COL1A2*—Figure 1). Further studies in recent years have identified new mutations in collagen-related genes, leading to the identification and classification of different types of OI and related genes, known as the “Sillence classification” (Sillence et al., 1979), which was recently updated with genetic classification. Thus, a correct diagnosis of OI can be obtained only after genetic investigation (Table 1).

**FIGURE 1:**
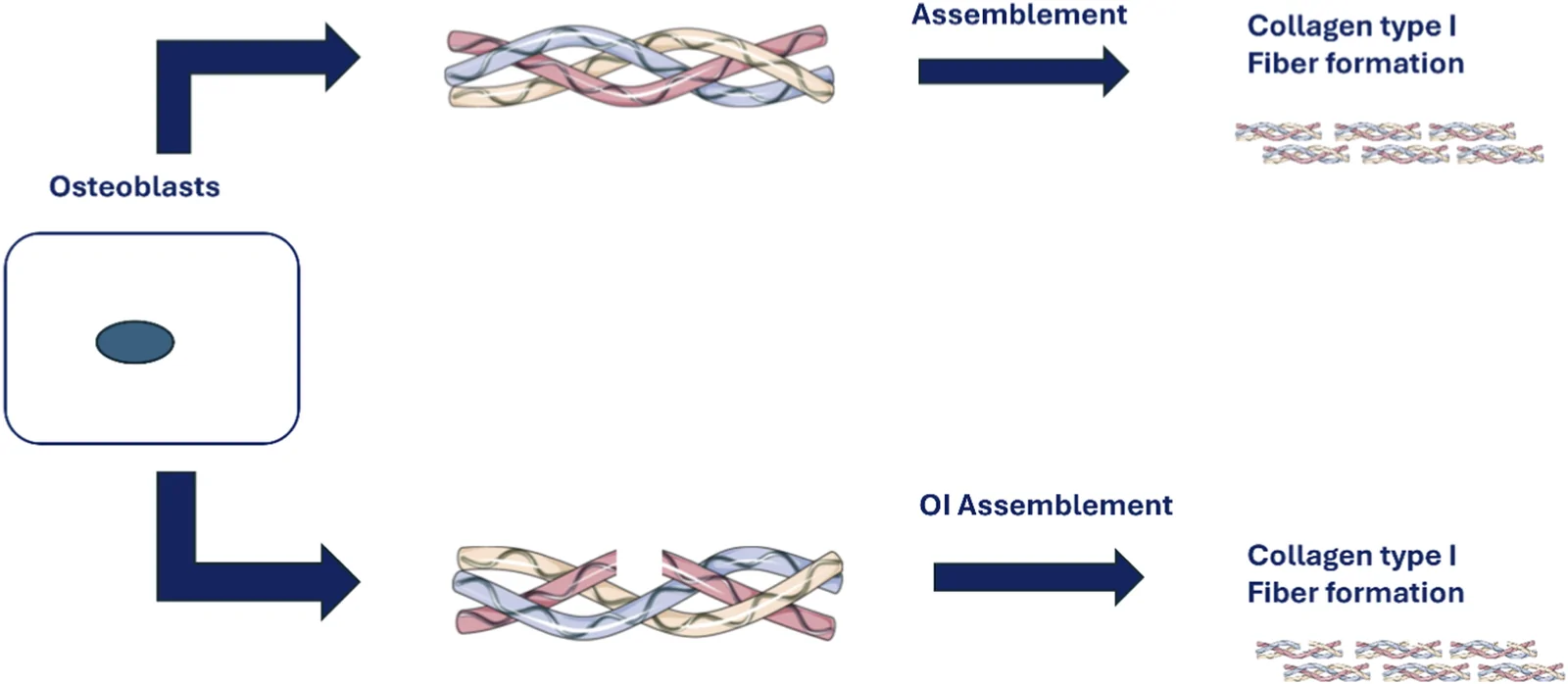
Collagen type 1 synthesis in controls and OI patients.

**TABLE 1:** Osteogenesis imperfecta genetic classification.

Type of osteogenesis imperfecta	Mutated gene	Encoded protein
I, II, III, IV	*COL1A1* or *COL1A2*	Collagen α1(I) (COL1A1) or (I)COL1A2)
V	*IFITMS*	Bone-restricted interferon-induced transmembrane protein-like protein (BRIL; also known as IFM5)
VI	*SERFINF1*	Pigment epitelium-derived factor (PEDF)
VII	*CRTAP*	Cartilage-associated protein (CRTAP)9
VIII	*P3H1* (previously known as *LEPRE1*)	Prolyl 3-hydroxylase-1 (P3H1)
IX	*PPIB*	Peptidyl-prolyl-cis-trans isomerase B (PPIase B)
X	*SERPINH1*	Serpin H1 (also known as HSP47)
XI	*FKBP10*	65 kDa FK506-binding protein (FKBP65)
No phenotype	*PLOD2*	Lysyl hydroxylase 2 (LH2)
XII	*BMP1*	Bone morphogenetic 1 (BMP1)
XIII	*SP7*	Transcription factor SP7, also known as “osterix”
XIV	*TMEM38B*	Trimeric intracellular cation channel type B (TRIC-B, also known as TM38B)
XV	*WNT1*	Proto-oncogene Wnt-1 (WNT1)
XVI	*CREB3L1*	Old astrocyte specifically induced substance (OASIS, known as CR3L1)
XVII	*SPARC*	SPARC, also known as “osteonectin”
XVIII	*MBTPS2*	Membrane bound transcription factor site 2 protease (S2P)
XIX	*TENT5A*	FAM46A
XX	*MESD*	MESD
XXI	*KDELR2*	KDEL RECEPTOR 2
XXII	*CCDC134*	CCDC134
XXIII	*PHLDB1*	PHLDB1

## Fibrillogenesis

The main protein present in the extracellular matrix of bone and body is type I collagen. The precursor is type I procollagen heterotrimer, which results from the assembly of two pro-α1 chains and one pro-α2 chain, synthetized in the ER and subjected to different post-translational modifications at proline and lysine thanks to prolyl-4-hydroxylase 1 (P4H1) and lysyl hydroxylase (LH1). Additional mediators work on the correct folding of procollagen: ER-residing prolyl 3-OH complexes (formed by P3H1, CRTAP, and PPIB), which hydroxylates the P896 amino acid fundamental for pro-collagen chain alignment. Together these complex ER chaperones support precise pro-collagen assembly and folding. The first is HSP47 that cooperates with FKBP10, which is fundamental for trafficking procollagen from ER to Golgi. KDEL receptor 2 participates in the recycling of resident proteins in ER by the retrograde transport. Ca^2+^ is important for post-translational modifications, and the calcium flux from ER to cytoplasm is regulated by TRIC-B and IP3R. Interestingly, TRIC-B positively affects mitochondrion fusion and fission. Old astrocyte specifically induced substance (OASIS) is another important transcriptional factor that moves from ER to Golgi for RIP signaling. Following its cleavage by S1P and S2P, the OASIS N-terminal end enters the nucleus, activating collagen gene transcription. Its polyadenylation is supported by FAM46A. The inhibition of ERK and JNK by CCDC34 impacts collagen synthesis. Following exocytosis, the procollagen triple helix undergoes C- and N-terminal cleavage by BMP1 and ADAMTS-2, respectively, to become mature type I collagen that participates in the matrix to form fibers thanks to different molecule assembly. In this phase, SPARC involvement is fundamental. Collagen fibers and fibrils are stabilized by the formation of crosslinks, with the involvement of lysine and hydroxylysines in the triple helix that are oxidized by lysyl oxidases to obtain allysine and hydroxyallysine. These initially interact to form divalent crosslinks that become mature trivalent pyridinoline- and pyrrole-stabilizing fibril structures.

## OI type

OI includes types I, II, III, and IV (autosomal dominant inheritance) with the impairment of collagen synthesis and structure, in which the mutated genes are COL1A1 or COL1A2, encoding for collagen α1(I) (COL1A1) or α2(I) (COL1A2) proteins with the classic phenotype. Type I OI patients show near-normal stature, blue sclera, and late-onset of hearing loss. Type II OI is a perinatally lethal form. Type III OI patients show a progressive deformity variety. Type IV OI patients report bone deformity which is more severe dentinogenesis imperfecta than type I OI but less than types II and III. In types II and III OI, diaphyseal long bone cortical tissue has been studied using light, polarization, and ultrastructural microscopy, showing that lamellar woven bone synthesis is normal but that maturation is delayed due to abnormal or insufficient collagen matrix (Shapiro et al., 2021). Despite a structurally stronger lamellar arrangement being evident when the correct deposition of bone matrix was reached, lamellar bone displayed short, obliquely oriented lamellae. Polarization defines tissue conformations and localizes the initiation of lamellar formation. Osteoblast ultrastructure shows evidently dilated rough endoplasmic reticulum (RER) and prominent Golgi bodies, with disorganized cisternae and swollen dispersed tubules and vesicles, signifying that storage disorder/stress responses are present. If mitochondrial swelling is also evident, the cell is in apoptosis. Ultrastructural matrix assessments in woven bone show randomly oriented individual fibrils but also short pericellular bundles of parallel oriented fibrils positioned obliquely and oriented randomly to one another; in lamellar bone, they show unidirectional fibrils that deviate at slight angles to adjacent bundles and obliquely oriented fibril groups consistent with twisted plywood fibril organization. Histomorphometric indices show that woven bone is more cellular than lamellar. Pericellular matrix accumulations in woven OI are the smallest, and normal lamellar are the largest.

Patients with types V and VI OI show compromised bone mineralization due to mutation in *IFITM5* (autosomal dominant inheritance) and *SERPINF1* (autosomal recessive inheritance), respectively. The *IFITM5* gene encoded for bone-restricted interferon-induced transmembrane protein-like protein (BRIL, also known as “IFM5”) leads to normal to severe skeleton deformity, radiodense band and radial head dislocation, intraosseous membrane ossifications, variable hearing loss, and normal to blue sclera. BRIL is highly expressed during osteoblast differentiation, especially during mineralization (Moffatt et al., 2008). In OI type V biopsies, although BRIL expression is similar to controls, augmented *in vitro* mineralization was evident (Reich et al., 2015). Interestingly, BRIL proteins and transcripts were similarly measured in bone from a patient with OI type V, suggesting that OI causes a gain in function involving incorrect intracellular activity of the mutant BRIL (Patoine et al., 2014). A new model was realized to better characterize OI type V, considering that others previously obtained gave embryonic lethality that was inducible in a mouse model (*Ifitm* ^*5floxc.−14C>T*^) (Tan et al., 2025). These mice had pups with fractures in all limbs with impaired ossification due to SOX9 overexpression. Decreased levels of SOST and SP7 were found with retarded osteocyte maturation and elevated adipocyte number. Using another model of type V OI—a conditional *Rosa26-*knockin mouse model—it was found that osteo-chondroprogenitor or chondrogenic cells resulted in low bone mass and growth retardation with impaired endochondral ossification, irregular growth plate structure, and cartilage overgrowth resembling the phenotype of type V OI (Marom et al., 2024). Additionally, chondrogenic cells had a reduced potential to differentiate in osteoblasts at diaphysis—altered due to the activation of ERK signaling and SOX9 consistently targeting these pathways; rescue was evident.

In contrast, the *SERPINF1 gene* encoded for pigment epithelium-derived factor (PEDF) with the consequent development of skeletal deformity in type VI OI that can appear as a moderate or severe form, with the presence of an osteoid, fish scale appearance lamellar bone pattern with onset in childhood. Hyper-mineralized bone is evident, although it is surrounded with low mineral content and elevated osteocyte number (Fratzl-Zelman et al., 2015), whereas circulating alkaline phosphatase levels are high (Patoine et al., 2014). PEDF is an angiogenesis inhibitor which binds collagen I in bone matrix (Dawson et al., 1999). Consistently, PEDF-collagen I interaction activates the inhibitory role of PEDF on angiogenesis; the inactivation of interaction sites leads to the neutralization of the effect on angiogenesis (Meyer et al., 2002; Hosomichi et al., 2005). In *Serpinf1* ^−/−^ mice, PEDF stimulates bone formation and inhibits vascularization (Bogan et al., 2013). Interestingly, in type IV OI, TGF-β signaling is activated, whereas Serpinf1^−/−^ osteoblasts are more responsive to TGF-β treatment than wild-type cells. TGF-β stimulation and PEDF deficiency show combined action on the osteogenic marker transcription suppression and pro-angiogenic factor stimulation. Additionally, PEDF reduced TGF-β-induced expression of pro-angiogenic molecules (Kang H. et al., 2022).

OI types VII, VIII, and IX (all autosomal recessive inheritance) are characterized by abnormal post-translational modification of collagen due to the different genes involved that are implicated in procollagen folding through the ER-residing prolyl-3OH complex, consisting of P3H1, CRTAP and PPIB. In this, mutations of the *CRTAP* gene encoding for cartilage-associated protein (CRTAP) lead to strong rhizomelia with white sclera typical of type VII OI. CRTAP is an essential component of a heterotrimeric, ER resident complex responsible for both collagen prolyl 3-hydroxylation and chaperone function. Importantly, human mutations in the *CRTAP* gene have been associated with recessive forms of OI, and *Crtap* ^−/−^ mice developed osteo-chondrodysplasia (Morello et al., 2006), characterized by reduced osteoid formation and severe osteopenia (Morello and Rauch, 2010).

Mutations in *P3H1-* (previously known as *LEPRE1*) encoding prolyl 3-hydroxylase 1 (P3H1) lead to type VIII OI, whereas mutation in the *PPIB* gene encoding for peptidyl-prolyl cis–trans isomerase B (PPIase B) leads to the development of type IX OI characterized by severe bone deformity and gray sclera. P3H1 functions in the extracellular matrix and the ER/Golgi compartments via its RGD (Arg-Gly-Asp) cell adhesion domain and a KDEL (Lys-Asp-Glu-Leu) ER retention sequence, respectively (Marini et al., 2010), (Takagi et al., 2012). The absence of P3H1 leads to the over-modification of type I collagen chains (Cabral et al., 2007). Mutations in *CRTAP* and *P3H1* lead to an abnormal 3-hydroxylation complex, post-translational modification defects, and structural abnormalities of collagen (Baldridge et al., 2008; Pokidysheva et al., 2013).

Other types of OI are characterized by compromised collagen processing and crosslinking, all with autosomal recessive inheritance. Mutations in the *SERPINH1* gene lead to altered serpin H1 (also known as HSP47) activity with the development of type X OI showing blue sclera, strong skeletal deformity, inguinal hernia, skin abnormalities, and dentinogenesis imperfecta. HSP47, a collagen-specific chaperone residing in the ER that simplifies collagen chain folding, binds to procollagen in ER and detaches from this in cis-Golgi, thus blocking the aggregation of procollagen (Bonfanti et al., 1998). *HSP47* ^−/−^ died in embryo (Nagai et al., 2000) due to deficiency in the maturation of collagen types I and IV as well as thin and frequently branched fibrils of collagen type I (Ishida et al., 2006).

Additionally, mutations in the *FKBP10* gene encoding for 65 kDa FK506-binding protein (FKBP65) lead to the diagnosis of type XI OI, which is characterized by normal to gray sclera, mild to severe deformity of the skeleton, and progressive contractures of the joint (Alanay et al., 2010; Kelley et al., 2011). FKBP65, a molecular chaperone residing in ER, binds collagen modulating its cross-linking (Ishikawa et al., 2008; Barnes et al., 2012). However, mutations of the *PLOD2* gene encoding for lysis hydroxylase (LH2), responsible for collagen stability dependent on inter-molecular cross-links realized through lysine hydroxylation, lead to a non-classified type of OI with skeletal deformity and joint contractures due to the under- and over-hydroxylation of collagen telopeptide lysine (Lim et al., 2017; Ha-Vinh et al., 2004).

Type XII OI is characterized by the mutation of the *BMP1* gene, which encodes for metalloprotease bone morphogenetic protein 1 (BMP1), clinically manifesting skeletal (mild to severe) deformity together with umbilical hernia. BMP1 together with an enhancer protein affects the cleavage of type I collagen pro-peptide processing and cross-linking (Martinez-Glez et al., 2012).

An additional group of OI is characterized by gene mutations that cause altered osteoblast activity and differentiation: OI types XIII-XVIII. Here, the mutation of the *SP7* gene (autosomal recessive inheritance), giving the key transcription factor SP7 (also known as “osterix”) with a key role in bone formation (Nakashima et al., 2002), characterizes type XIII OI that is phenotypically characterized by facial hypoplasia, skeletal deformity, and delayed eruption of teeth. Mutations of the *TMEM38B* gene that encodes for the ER membrane trimeric intracellular cation channel type B (TRIC-B; also known as TM38B) lead to type XIV OI (autosomal recessive inheritance), which is phenotypically characterized by normal-to-blue sclera and severe bone deformity.

TRIC-B works with inositol 1,4,5-triphosphate receptor (IP3R) to regulate Ca^2+^ flux from ER to cytoplasm (Yazawa et al., 2007). Ca^2+^ is important for collagen metabolism. An impaired ER-Ca^2+^ flux has been consistently found in *TMEM38B* ^−/-^ fibroblasts and osteoblasts with consequent ER stress and altered collagen I synthesis and secretion (Yazawa et al., 2007). The lack of TRIC-B determined a decrease in the lysine hydroxylation of the collagen helical region and an increase in the hydroxylation of telopeptides. Consequently, the assembly of the pro-collagen chain was delayed, and thus also the final molecule in the extracellular matrix (Cabral et al., 2016). In osteoblasts, the lack of TRIC-B impaired bone differentiation and mineralization due to the prolonged ER stress (Jones et al., 2025) was also determined by the elongation of mitochondria due to the reduced expression of mitochondrial fusion/fission markers. These organelles also increased the production of ROS, and OI osteoblasts consistently showed mitochondria changes (Jovanovic et al., 2023).

The mutation of the *WNT1* gene that encodes for the proto-oncogene Wnt-1 (WNT1), typical of type XV OI (both autosomal dominant or recessive inheritance), leads to possible neurological defects, white sclera, and strong skeletal abnormalities (Laine et al., 2013). *Wnt1* deletion in osteocytes determines low bone mass with spontaneous fractures; conversely, *Wnt1* overexpression from osteocytes promotes bone formation by augmenting osteoblast number and activity (Joeng et al., 2017). Biallelic nonsense mutations or frameshift mutations of *WNT1* could lead to an earlier occurrence of fragility fractures and a more severe skeletal phenotype in OI (Hu et al., 2023). Tan et al. (2024) reported that multi-omics analyses reveal an aberrant differentiation trajectory with WNT1 loss-of-function in type XV OI. However, the *CREB3L1* gene encoding for old astrocyte specifically induced substance (OASIS, also known as “CR3L1”) diagnoses, as type XVI OI is characterized by severe bone deformity with autosomal recessive inheritance. OASIS binds to the gene promoter *Col1a1*, increasing its transcription. *OASIS* ^−/−^ mice consistently developed osteopenia and fractures due to the reduced collagen type I levels in bone (Murakami et al., 2009). Mutation in SEC24D encoding for a protein of the COPII-dependent ER to Golgi transfer with consequent retention of procollagen in ER consequently caused patients display gray-blue sclera, Wormian bones, and shortened or bent long bones (Garbes et al., 2015). *SEC24D* mutation inhibits osteoblastogenesis, and it has been reported that the TGF-β pathway impairs and mediates this effect through ATF6 (Zhang et al., 2025).

Type XVII OI (autosomal recessive inheritance) is diagnosed by the identification of mutations of the *SPARC* gene, encoding for SPARC protein—osteonectin—which phenotypically leads to severe bone alterations (Storoni et al., 2023). SPARC is a glycoprotein that binds different proteins of the extracellular matrix, including collagen, and it is fundamental for bone mineralization (Durkin et al., 2022; Termine et al., 1981). *SPARC* ^−/−^ mice showed osteopenia due to reduced bone formation, and osteoblasts lead to reduced bone remodeling (Delany et al., 2000).

Furthermore, mutations of the *MBTPS2* gene that encodes for membrane-bound transcription factor site-2 protease (S2P) have been diagnosed as type XVIII OI X-linked recessive, displaying light blue sclera, skeletal deformities, pectoral deformity, and scoliosis. Danyukova et al., 2023 realized two *Mbtps2* mouse models—a knock-in mouse carrying one of the disease-causative *MBTPS2* variants (*N455S*) and an *Mbtps2* ^−/−^mouse. These mice showed a phenotype resembling early osteoarthritis with osteochondral abnormalities characterized by thinned subchondral bone, altered subchondral osteocyte interconnectivity, thickened articular cartilage with chondrocyte clustering, and without alteration of bone turnover and mass.

After 2006, the Sillence classification gave rise to various advances due to the identification of new genes that cause OI, culminating in the Genetic Classification, which included 22 OI types (Jovanovic and Marini, 2024). The newly classified OI types included XIX, XX, XXI, and XXII, all with autosomal recessive inheritance. Type XIX OI is due to a mutation *TENT5A* encoding for FAM46A protein, a member of the nucleotidyl-transferase (NTase)-fold protein family of noncanonical poly(a) polymerases (Gewartowska et al., 2021) that cause a lethal to severe form of OI with defects in the BMP/TGFβ signaling pathway. Type XX OI is due to mutation of the *MESD* gene that encodes for the homonym protein, MESD—a chaperone residing in the endoplasmic reticulum (ER) that translocates LRP5 and LRP6 (Hsieh et al., 2003). MESD disfunction in a patient’s cells thus leads to the disruption of WNT signaling. Type XXI OI is characterized by mutation in *KDELR2* which encodes KDEL receptor 2, leading to a severe progressive form of OI with multiple fractures, short stature, and chest deformity. KDELR2 is a member of the KDEL receptor family, localized in ER, the intermediate ER-Golgi compartment, and cis-Golgi. These receptors form the COPI vesicles that work in the retrograde transport of ER-resident proteins that contain the peptide Lys-Glu-Asp-Leu (KDEL) from the cis-Golgi to the ER (Capitani and Sallese, 2009; Brauer et al., 2019). KDELR2 loss of function determines the normal secretion of HSP47 in the extracellular matrix; however HSP47 interaction with collagen molecules in the matrix lead to impaired fibrillogenesis with the formation of thin fibrils (van Dijk et al., 2020). XXI OI fibroblasts consistently showed decreased intracellular and secreted Collagen I. *Kdelr2* ^−/−^ mice experienced premature death, with altered skeleton phenotype and cleft palate (Dickinson et al., 2016).

Type XXII OI is characterized by the *CCDC134* gene mutation that encodes for the CCDC134 protein, leading to a severe form of OI with short stature, multiple fractures, reduced bone mineral density, bowing, and Wormian bones. It develops as a consequence of the dysregulated MAPK/ERK pathway because CCDC134 is involved in the MAPK pathway, the inhibition of JNK, and ERK phosphorylation (Huang et al., 2008). *In vitro* osteoblasts from type XXII OI have consistently shown augmented ERK1/2 phosphorylation, decreased osteopontin, collagen I, and mineralization (Dubail et al., 2020). In 2023, a biallelic pathogenic variant of pleckstrin homology-like domain family B member 1 (*PHLDB1*) was described as mild OI and was registered as type XXIII OI in OMIM (Tuysuz et al., 2023). *PHLDB1* is involved in multiple phosphorylation processes. It has a autosomal recessive inheritance, and patients have reported osteopenia, recurrent fractures, and short and bowed long bones.

## Additional mediators of OI

Both human and murine *in vivo* and *in vitro* models have been used to identify potential new targets for OI management. The role of Schnurri-3 has been demonstrated using an *oim/oim* mouse model of OI (Li et al., 2024). This is a fundamental negative modulator of bone formation both in humans and mice. *Schnurri-3* ^*−/−*^ showed osteosclerotic bone due to increased osteoblast activity, thus offering protection from bone loss associated with aging (Jones et al., 2006; Morris et al., 2019). This molecule also stimulates the production in osteoblasts of the pro-angiogenic factor SLIT3 (Li et al., 2020; Kusumbe et al., 2014). *Schnurri-3* ^*−/−*^ mice consistently display augmented skeletal vascular endothelium. These findings led to inducing *Schnurri-3* ^*-*^ knockdown in an *oim/oim* mouse model of OI with the consequent rescue of bone impairment, thus supporting its use as a frontier OI management.

*The oim/oim* mouse model has also been used to study the role of immune cells in bone disease that characterizes OI. Kang I. H. et al. (2022) reported that T cells in this model showed an activated phenotype together with an elevated secretion of IFNγ and TNFα, with natural spleen and thymus Tregs decreased. TGF-β receptor 1 and II expressions were found to be decreased in splenic T cells of OI mice. Furthermore, Kang I. H. et al. (2022) reported that conditioned media from wild-type and OI mice inhibited osteoclastogenesis and increased osteoblast mineralization. Interestingly, OI mice transplanted with Treg from WT mice displayed an improved bone phenotype due to the reduced T cell activation and cytokine production, with consequent decreased osteoclasts and increased osteoblasts.

TGFβs are secreted as a complex with its pro-domain latency associated peptide (LAP), which confers latency, either altering TGFβ confirmation or blocking receptor access (Wu et al., 2024). TGFβs can also be secreted as a complex of LAP and latent TGFβ-binding protein (LTBP), leading to Large Latent Complex (LLC). Furthermore, TGFβ LLC interacts with LTBP and fibrillin-1. Correct TGFβ signaling is strictly related to the absence of a genetic problem in LTBP, LAP, and fibrillin-1. The interaction of TGFβs with their receptors has led to the phosphorylation of the downstream mediators SMAD2/3 that, following the interaction with Smad4, translocate in the nuclei to activate specific transcription factors (Shi and Massague, 2003). The embryonic ablation of osteoblast *Smad4* consistently reproduced an OI-like phenotype (Salazar et al., 2013). LTBP ablation with consequent decreased bioavailability of TGFβ leads to bone disease (McInerney-Leo et al., 2016; Dabovic et al., 2005). In a different model, TGFβ involvement has been demonstrated in *Crtap* ^−/−^ (recessive OI), *Col1a2* ^+/G610C^ mice (dominant OI), and *Brtl* ^+/−^ mice (resembling OI type IV) (Forlino et al., 1999; Bianchi et al., 2015), as well as *Col1a1* ^+/Jrt^ mice (dominant OI) (Tauer et al., 2019). TGFβ involvement in OI led to altered interaction/signaling with the bone matrix components as decorin (Grafe et al., 2014). However, TGFβ in OI is important and consistently represents a new target to counteract this disease.

Another important axis involved in OI is represented by the RANKL/RANK/OPG/NFκB signal (Etich et al., 2020a). OPG is secreted by osteoblasts, osteocytes, and immune cells that inhibit osteoclastogenesis (Simonet et al., 1997). OPG is a decoy receptor for a receptor activator of nuclear factor κ B ligand (RANKL); following their interaction, the binding with its cognate receptor (RANK) is blocked on the osteoclast membrane, thereby inhibiting their formation and bone resorption activity (Fuller et al., 1998). RANKL is mainly secreted by osteocytes (Nakashima et al., 2011; Xiong et al., 2011) and also by osteoblasts and immune cells. RANKL/RANK binding allows the recruitment of TNF-receptor-associated factor (TRAF) adaptor proteins to the TRAF domain contained in the cytoplasmic tail of RANK (Darnay et al., 1998). TRAF6 is the most important for osteoclast formation and function consistency; *TRAF6* ^−/−^ mice display severe osteopetrosis due to altered osteoclastogenesis (Lomaga et al., 1999; Naito et al., 1999). TRAF6 activates the nuclear factor kappa B (NFκB), c-Jun N-terminal kinase (JNK), ERK, p38, AKT, and NFATc1 (Matsumoto et al., 2000; Wong et al., 1999; Takayanagi et al., 2002). It has been reported that RANKL signaling modulates osteoblastogenesis (Chen et al., 2018), where RANK ablation in bone marrow mesenchymal stem cells (BMSCs) expressing paired-related homeobox 1 (Prx1) or overexpression or silencing led to the activation of forward RANKL signaling, thus inhibiting osteoblastogenesis from BMSCs—principally by activating NFκB and inhibiting β-catenin synthesis/degradation (Chen et al., 2018). RANK was found to be released from the differentiated osteoclasts through extracellular vesicles (Huynh et al., 2016) to bind RANKL on osteoblast membrane to activate reverse signal transduction *in vitro* and *in vivo*. Furthermore, upon RANKL multimeric interaction through a characteristic proline-rich motif in the RANKL cytoplasmic tail with Src homology 3 (SH3), domains of Src family kinases stimulate the PI3K-AKT–mTOR pathway and thus RUNX2 (Zhang et al., 2017; Ikebuchi et al., 2018).

Abnormal RANKL and OPG levels have been observed in non-lethal *Brtl* ^+/−^ mice carrying a G349C substitution in *Col1a1* and *oim/oim* mice harboring a mutation in *Col1a2* (Uveges et al., 2008; Li et al., 2010). In addition, increased RANKL serum and mRNA levels but reduced OPG mRNA levels in lymphomonocytes have been observed in 18 pediatric OI patients classified as OI types I, III and IV, supporting the high osteoclastogenic potential of patients’ peripheral blood mononuclear cells in multiple OI forms (Brunetti et al., 2016). In primary osteoblasts, OPG expression has been described as regulated by PEDF that is encoded by the *SERPINF1* gene, thus inhibiting osteoclast function (Akiyama et al., 2010), besides its role in inducing β-catenin signaling and regulating sclerostin expression. PEDF also induced the osteogenic differentiation of mesenchymal stem cells and osteoblast mineralization via the ERK and AKT signaling pathways (Li et al., 2013). The mechanism by which PEDF regulates OPG expression is still incompletely understood but implies that loss of functional PEDF leads to an activation of osteoclasts via the RANKL/RANK/NFκB pathway in OI patients with *SERPINF1* mutations.

In OI types I, III, and IV, increased RANKL and decreased OPG messenger RNA amounts were demonstrated in lymphomonocytes from patients, together with high TNF-α levels in monocytes and an elevated proportion of circulating CD11b-CD51/CD61+ osteoclast precursors; higher RANKL levels were also measured compared with controls (Brunetti et al., 2016). The same study also demonstrated the correlation of OPG and RANKL levels with the amounts of DKK1, an osteoblastogenesis inhibitor of the WNT-pathway.

Cytoskeleton and focal adhesion structural aberrations have been found in collagen type I-secreting cells of the *Brtl* ^+/−^ OI model (Bianchi et al., 2015), possibly due to altered integrin-mediated cell-collagen binding, leading to impaired cell activity. Aberrant cyto- and nucleo-skeletons in fibroblasts were found in patients with milder, recessive OI forms (types VII, VIII, and IX) caused by mutations in the genes encoding *CRTAP*, *P3H1*, and *PPIB*, respectively (Gagliardi et al., 2017).

## Osteocytes

Osteocytes are long-lived bone cells and are the most numerous, representing the mechanosensory of the bone and regulating osteoclast and osteoblast activity. OI bone has an increased osteocyte density in human and murine models (Chipman et al., 1993; Zimmerman et al., 2018), and these cells are embedded in a matrix rich in abnormal type I collagen. Zimmerman et al. (2018) hypothesized that osteocyte-derived RANKL contributes to the OI bone phenotype. In this study using *oim/oim* mice, the conditional loss of RANKL in osteocytes (oim-RANKL-cKO) resulted in severely increased trabecular bone mass in both the femur and lumbar spine with respect to control *oim/oim* mice. Bone cortical thickness only augmented significantly in the spine, but bone strength in the long bones and spine was modestly improved in oim-RANKL-cKO mice compared to *oim/oim* mice. The lack of osteocyte-derived RANKL further reduced bone formation parameters in oim-RANKL-cKO, indicating that osteocytes significantly contribute to the low bone mass observed in OI, and the effect of RANKL loss from osteocytes is comparable to its systemic inhibition.

*In vivo* transcriptional changes in osteocytes from *Crtap*KO and *oim/oim* murine models of OI compared with wild-type (WT) control mice demonstrated that a large transcript number was dysregulated in both models, but 281 of them were similarly up- or downregulated with respect the WT controls (Zimmerman et al., 2019). Otherwise, very limited transcripts were diversely expressed between the *Crtap*KO and *oim/oim* mice, implying that distinct aberrations in type I collagen can generate common pathogenic processes and comparable phenotypic outcomes. Bioinformatic analyses identified their involvement in development and differentiation, extracellular matrix and collagen fibril organization, cell adhesion, signaling, regulatory processes, pattern binding, chemotaxis, and cell projections. The results highlighted alterations in the WNT and TGFβ pathways but also highlighted new candidate genes to pursue in future studies. Mahr et al. (2021) evaluated 2D osteocyte lacunae sections (OLS) based on quantitative backscattered electron imaging in trans-iliac bone biopsy samples from children with OI type I and age-matched controls. They also evaluated pediatric bone mineralization density distribution in OI type I and controls using a field emission scanning electron microscope. OI patients showed an augmented OLS density in both trabecular and cortical bones, but otherwise the OLS area was reduced in the trabecular bone. Additionally, Mahr et al. (2021) found a low to moderate positive correlation of OLS density with surface-based bone formation parameters together with a negative correlation with indices of osteoblast activity.

## Muscle OI

OI also presented strong deficits in numerous features of skeletal muscle function, including lower muscle strength and power, greater fatigability, and impaired balance which derived from reduced muscle mass and poor muscle quality (Ireland et al., 2025). These alterations have fundamental implications for different traits of health and general function, including mobility, fracture risk and fall, and the competence to perform daily activities. In OI, the weakness and impaired activity of muscle is initially multi-factorial, and factors such as deficiency in ventilatory, sensory, and metabolic function may reflect muscle mass and quality. Mitochondrial dysfunction has been demonstrated in the skeletal muscle of *oim/oim* mice. Decreased mitochondrial respiration in the female gastrocnemius muscle of *oim/oim* mouse has been reported. Myosin heavy-chain fiber-type distribution is abnormal in the *oim/oim* soleus muscle with a reduction (−33%–50%) in type I myofibers and an increase (+31%) in type IIa myofibers compared to their wildtype littermates. Furthermore, augmented energy expenditure and different body composition were found in *oim/oim* mice compared to WT mice, suggesting that the mitochondrial dysfunction of the skeletal muscle correlated with the metabolic alterations of the whole body and with the weakness of skeletal muscle in *oim/oim* mice (Gremminger et al., 2021). The synergistic biochemical and biomechanical relationship between bone and muscle is a critical potential therapeutic target, such that muscle weakness should not be ignored. Consistently, studies in OI patients demonstrate altered energy metabolism, mechanosensing, mitochondrial dysfunction, and paracrine/endocrine crosstalk in OI pathogenesis. The altered muscle–bone unit affects mechanosensing and the ability of OI muscle and bone to respond to therapy to improve the health of both tissues (Phillips and Jeong, 2018).

Myostatin and activin A, members of the TGFβ superfamily, inhibit muscle growth by binding the activin receptor (Lee et al., 2005; Latres et al., 2017). Heterozygous *+/oim*, a mild OI model, bred with *myostatin* ^−/−^ mice generate mice with increased skeletal bone and muscle (Oestreich et al., 2016). Different murine OI models showed a similar muscle phenotype of human OI, and treatment with activin receptor 2B, inhibiting both activin A and myostatin, led to a beneficial effect on the bone of *oim/oim*, *Col1a2* ^+/G610C^, and *Col1a1* ^+/Jrt^ mice (DiGirolamo et al., 2015; Tauer and Rauch, 2019; Jeong et al., 2018).

## Therapy

Currently, in the guidelines for OI management referring to bisphosphonates, neridronate is the only one for pediatric age (Gatti et al., 2005), whereas others can be used in adulthood (Dwan et al., 2016; Ma et al., 2017). However, monoclonal antibodies against RANKL (Hoyer-Kuhn et al., 2014), TGFβ (Song et al., 2022), and sclerostin have also used. However, the use of anabolic agents, including parathyroid hormone analogs (Gatti et al., 2013), stimulate the formation of new bone matrix with impaired quality. The new frontiers for OI management are gene and cell therapy. Physical activity must always be associated with a therapeutic approach to improving bone health in OI.

### Bisphosphonates

Bisphosphonates inhibit bone resorption by osteoclasts and stimulate their apoptosis with a consequent increase in BMD, decrease in pain, and augmented muscle strength. They do not impact bone growth and modeling, but their prolonged use could generate adverse effects. Bisphosphonate treatment has led to the accumulation of a thin sclerotic band of mineralized cartilage between the metaphysis and the growth plate as well as decreased osteoclast activity. This therapy did not enhance bone mass in OI children and *oim* mice. D'Eufemia et al. (2017) demonstrated that the cumulative effect of neridronate administration increases serum levels of brain isoenzyme of creatine kinase (CK-BB), expressed by mature osteoclasts, and the reversible effect after its discontinuation. This approach was used to verify the usefulness of serum CK-BB as a biochemical marker in patients receiving prolonged bisphosphonate treatment. These CK-BB levels increased in the serum of OI-treated patients through its secretion by osteoclasts undergoing apoptosis (Faienza et al., 2023).

### Denosumab

Denosumab is a monoclonal antibody that targets RANKL, thus decreasing the differentiation, activity, and survival of osteoclasts (Etich et al., 2020b); it has been approved for primary osteoporosis treatment (Majdoub et al., 2023). A very recent study revealed that denosumab not only significantly increased areal BMD (aBMD) in children with OI but also ameliorated spinal morphometry, with rebound hypercalcemia as an adverse effect (Liu et al., 2024; Trejo et al., 2018). Denosumab and zoledronic acid were used in a year-long study for adult OI management, showing that treatment with denosumab significantly enhanced aBMD at the lumbar spine and total hip and markedly decreased bone resorption in adult patients with OI (Lin X. et al., 2024). Additionally, denosumab efficacy was comparable to that of zoledronic acid, but denosumab treatment resulted in fewer adverse effects than the latter.

### Anti-sclerostin antibodies

Different OI murine models have been used to evaluate the effect of sclerostin inhibition, reporting an improvement of bone quality (reviewed in Misof and Fratzl-Zelman, 2024). It is not recommended for OI children; in adult OI, it has been used in a few patients, increasing bone mass together with serum bone formation markers (Glorieux et al., 2017; Uehara et al., 2022; Kusakabe et al., 2024).

Setrusumab, a neutralizing antibody to sclerostin, was used for adults with a clinical diagnosis of OI types I, III, or IV management for 1 year in the phase 2b Asteroid (NCT03118570) study (Glorieux et al., 2024). This trial reported a beneficial effect of setrusumab on OI bone strengths, sustaining its phase 3 evaluation. The ASTEROID phase-2b multicenter trial saw a dose-dependent increase in bone formation and resorption with setrusumab treatment that was modeling-based (Hosseinitabatabaei et al., 2025).

### Teriparatide

Teriparatide is the analog of recombinant human PTH, and clinical trials have demonstrated that bone resorption and formation markers together BMD were enhanced in OI, although the effect of bone markers is much more evident in type I OI than in types III/IV (Orwoll et al., 2014). The results from a recent trial involving 350 OI patients reported that teriparatide plus zoledronic acid did not reduce fracture risk compared with standard care, despite significantly increasing BMD; this suggests the importance of reduced bone quality rather than low bone density in the pathogenesis of fracture (Hald et al., 2026).

### Anti-TGFβ antibody

Anti-TGFβ treatment showed a beneficial effect on OI both in human and murine models, increasing bone formation and brittleness (Sun et al., 2023). The first-in-human phase I study using TGFβ antibody (fresolimumab) was demonstrated to ameliorate bone mass with increases in lumbar spine aBMD in adult patients with OI type IV (Song et al., 2022).

## Cell and gene therapy

An alternative approach for OI management looks to improve osteoblast activity by transplanting healthy cells or by silencing the mutant allele (Botor et al., 2021; Horwitz et al., 1999; Schindeler et al., 2022). Two clinical studies on the intravenous administration of histocompatible MSCs from donors to children with severe OI have been performed, with six and two children treated, respectively, where six children underwent myeloablative hematopoietic stem cell transplantation before they received MSCs (Horwitz et al., 2001; Horwitz et al., 2002; Infante et al., 2021). The MSC administration procedures were safe, no serious adverse events were registered, and positive clinical effects were attained with donor cell engraftment, improved bone parameters, and new compact bone developing; 6 months following two MSC infusions, there was an increase in growth velocity, an 80% reduction in the frequency of fractures, and an increase in total bone mineral content in three of the eight children (Horwitz et al., 2002; Infante et al., 2021) and in quality of life (Infante et al., 2022). Preclinical data and case studies report that multiple intravenous administrations of MSCs present promising outcomes in OI management. The “Boost to Brittle Bones” (BOOST2B) trial aimed to assess the safety and tolerability of multiple intravenous and intraosseous administrations of fetal liver-derived MSCs in severe OI children aged 1 to 5 years (Madhuri et al., 2025; https://inclinicaltrials.com/osteogenesis-imperfecta/NCT04623606/). In Europe, the “Boost Brittle Bones Before Birth” (BOOSTB4) trial evaluated the administration of allogeneic expanded human first trimester fetal liver MSCs (BOOST cells) for OI type III or severe type IV (Sagar et al., 2024). BOOSTB4 is an exploratory, open-label, multiple dose, phase I/II clinical trial to assess the safety and efficacy of the postnatal (n = 15) or pre- and postnatal (n = 3, originally n = 15) administration of BOOST cells for treating severe OI compared with a combination of historical (1–5/subject) and untreated prospective controls (≤30). Infants 18 months old (originally<12 months) and single pregnant women whose fetus had severe OI with confirmed glycine substitution in COL1A1 or COL1A2 were included in the trial. Each subject received four intravenous doses of 3 × 10^6^/kg BOOST cells at 4-month intervals, with 48 (doses 1–2) or 24 (doses 3–4) hours in-patient follow-up, primary follow-up at 6 and 12 months after the last dose, and long-term annual follow-up until 10 years after the first dose. Prenatal subjects received the first dose via ultrasound-guided injection into the umbilical vein within the fetal liver (16 + 0 to 35 + 6 weeks), and three doses postnatally. The primary outcome measures were safety and tolerability of repeated BOOST cell administration. The secondary outcome measures were number of fractures from baseline to primary and long-term follow-up, growth, change in bone mineral density, clinical OI status, and biochemical bone turnover. Further time is required to obtain results. Trial registration numbers are EudraCT 2015–003699-60, EUCT: 2023–504593-38–00, and NCT03706482. Importantly, these trials used fetal MSCs isolated from the first-trimester liver, which at this developmental stage is the blood-forming organ (Gotherstrom et al., 2004). Fetal MSCs show the same phenotype as adult MSCs and seem to have even lower immunogenicity, since adult MSCs express HLA class II antigens on the cell surface more quickly (<24 h) following interferon gamma (INF-γ) treatment, while fetal MSCs need approximately 7 days’ treatment to express HLA class II antigens on the cell membrane (Gotherstrom et al., 2004). Fetal MSCs have numerous additional positive properties over adult MSCs that are appropriate for their use in cell therapy; they are localized at an elevated frequency, show a major colony-forming capacity, are senesce delayed, and show greater proliferative and differentiation properties than adult MSCs (Zhang et al., 2009).

Novel genome editing technologies allow mutation-causing diseases to be directly corrected by reprogramming patient-derived fibroblasts into induced pluripotent stem cells (iPSCs) through viral or non-viral vectors (Vanleene et al., 2011; Fus-Kujawa et al., 2023; Lin C. et al., 2024). Here, fibroblasts derived from OI type II patients have been successfully reprogrammed into iPSCs using Yamanaka factors. Delivery of the correct linear DNA fragment to patient cells resulted in the repair of the DNA mutation, with an 84% success rate. iPSCs showed 87% viability after STAR treatment and 82% with its polyplex (Fus-Kujawa et al., 2023). To overcome the limitation associated to the use of a viral vector, alternative methods that bypassing this issue have been evaluated (Fernandes et al., 2008).

The most recent approach, Open New Horizon, employs gene editing using the CRISPR/Cas9 system, representing the most transformative genetic technologies that can be used in mammalian cells (Doudna and Charpentier, 2014; Shen et al., 2014). One example of its application is in Cao et al. (2023), who identified a novel cis-double-variant c.[175C>T; 187T>A] in *COL1A1* from a 5-year-old OI patient using whole-exome sequencing (WES). They cultured iPSCs from patient-derived fibroblasts to obtain iPSC clones with a c.187T>A variant that mainly restored their osteogenic properties after CRISPR/Cas9 gene editing. Importantly, correcting the c.187T>A variant alone in CRISPR-edited iPSCs was appropriate to alleviate OI phenotypes, as demonstrated by the enhanced amounts of *COL1A1*, *COL1A2*, *ALP* mRNAs, and COL1A1 proteins. These findings strongly encourage the use of CRISPR/Cas9 gene editing as a new therapeutic tool for OI management (Cao et al., 2023).

## Conclusions

OI is a genetic disease that appears with well-characterized different forms but requires new approaches in order to optimize its treatment. Until now, the use of bisphosphonates and/or monoclonal antibodies, teriparatide, represents elective therapy for management, but different trials are also ongoing using cell and gene therapies. At this time, all studies are searching for the right combination of drugs to decrease fractures in OI, although cell and gene therapies promise to treat this disease and improve quality of life.

Importantly, OI routine screening is limited to prenatal ultrasonography imaging that can identify perinatally lethal forms by 14–16 weeks of gestation. Type III can be identified by ∼18 weeks of gestation when longitudinal growth is close to the fifth percentile. The sequencing of chorionic villus samples can be utilized prenatally for OI diagnosis in subjects with OI family history. Pre-implantation genetic diagnosis is available for families with OI family history. For those cases, embryos deriving from *in vitro* fertilization can be screened, and those not at risk of developing the disorder can be selected. The utilization of eggs or sperm from an unaffected donor can be used to guarantee that parental OI is not transmitted.

OI types II and III are the forms most strongly associated with antenatal fractures, with type IV occasionally affected. In a study involving 540 OI patients, individuals with type III OI had the highest rate of at-birth fractures (92.6%), followed by those with types IV (50.7%) and I (17.2%) (Bellur et al., 2016).
